# Effect of anti‐attention‐deficit hyperactivity disorder (ADHD) medication on clinical seizures and sleep EEG: A retrospective study of Japanese children with ADHD

**DOI:** 10.1002/npr2.12215

**Published:** 2021-10-20

**Authors:** Hisako Yamamoto, Eiji Nakagawa, Yousuke Kita, Yoshimi Kaga, Masumi Inagaki

**Affiliations:** ^1^ Department of Child Neurology National Center Hospital National Center of Neurology and Psychiatry (NCNP) Kodaira Japan; ^2^ Department of Pediatrics St. Marianna University School of Medicine Kawasaki Japan; ^3^ Mori Arinori Center for Higher Education and Global Mobility Hitotsubashi University Tokyo Japan; ^4^ Cognitive Brain Research Unit (CBRU) Faculty of Medicine University of Helsinki Helsinki Finland; ^5^ Department of Developmental Disorders National Institute of Mental Health NCNP Kodaira Japan; ^6^ Department of Pediatrics Faculty of Medicine Yamanashi University Yamanashi Japan

**Keywords:** anti‐ADHD drugs, atomoxetine, attention‐deficit hyperactivity disorder, EEG, epilepsy, methylphenidate

## Abstract

**Aims:**

Patients with attention‐deficit hyperactivity disorder (ADHD) often exhibit basic or paroxysmal wave abnormalities on electroencephalography (EEG). Methylphenidate (MPH), an anti‐ADHD stimulant, has been reported to lower the seizure threshold. However, there have been no reports comparing EEG changes before and after administration of the central nervous system (CNS) stimulant MPH, or atomoxetine (ATX) hydrochloride, a non‐CNS stimulant. In this study, we investigated changes in sleep EEG before and after the administration of ADHD treatment drugs.

**Method:**

With the approval of the ethics committee, the medical records of 28 children with ADHD (23 men and 5 women) who gave consent were retrospectively investigated. The appearance of sudden abnormal waves during a 10‐minute sleep EEG recording was measured in 0.1‐second units, and the duration of these waves was calculated as the paroxysmal index (PI).

**Results:**

Paroxysmal index did not differ significantly between patients who received MPH and those who received ATX. In addition, there were no exacerbations of clinical seizures.

**Conclusion:**

It was concluded that ADHD medications do not have an adverse effect on epileptic seizures or abnormal sleep EEGs.

## Abstract 2

Patients with attention‐deficit hyperactivity disorder (ADHD) often exhibit basic or paroxysmal wave abnormalities on electroencephalography (EEG). We investigated changes in sleep EEG before and after the administration of ADHD treatment drugs. ADHD medications do not have an adverse effect on epileptic seizures or abnormal sleep EEGs.

## Graphical abstract

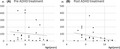

## 1 INTRODUCTION

Attention‐deficit hyperactivity disorder (ADHD) is a developmental disorder characterized by inattention, hyperactivity, and impulsivity. The rate of comorbid epilepsy in patients with ADHD is 2.6%‐27.7%, which is higher than in the general population.^1^, ^2^, ^3^, ^4^ In addition, the rate of comorbid ADHD in patients with epilepsy is as high as 30%‐40%,^5^, ^6^ indicating a close relationship between ADHD and epilepsy.

Furthermore, patients with ADHD often exhibit basic or paroxysmal electroencephalography (EEG) abnormalities. According to a study by Hughes et al,^7^ EEG findings in patients with ADHD showed that 27.8% of patients exhibited normal findings, whereas the abnormal findings included positive spikes, focal epileptic discharges, slow waves, extreme spindles, and bilateral spike‐and‐wave patterns. An increase in θ waves and a decrease in β waves are basic wave abnormalities; these waves were compared before and after treatment using the θ/β ratios.^8^, ^9^, ^10^ It is also known from a previous report^11^ that the distribution of basic waves changes with age. Some studies have failed to replicate θ/β ratios differences between ADHD and non‐ADHD.^12^, ^13^ Several studies have been conducted on these basic wave abnormalities, and it has been found that there are few α waves and many δ and θ waves with high amplitudes of slow waves and low amplitudes of α waves on EEGs performed in patients who were awake.^14^ Moreover, the high activity of θ waves remains until adulthood,^15^ suggesting cerebral dysfunction and immaturity in patients with ADHD.

Attention‐deficit hyperactivity disorder and autism spectrum disorders (ASD) are neurodevelopmental disorders, and both are onset in childhood. ASD and ADHD often co‐occur, with comorbidity rates in the 40%‐70% range.^16^ ASD is also often associated with EEG abnormalities. Reinhold et al^17^ reported that out of 316 children evaluated for ASD, 85 (27%) had EEG abnormalities, and Chez et al^18^ reported that 40 of 889 (60.7%) had EEG abnormalities. Another report^19^ evaluated EEG abnormalities with ASD were high rates as high as 85.8% (870/1014). In recent study,^20^ EEG abnormalities were found in 39.1% of patients, and EEG abnormalities correlated with autism severity, hyperactivity, anger outbursts, aggression, negative or destructive behavior, motor stereotypies, intellectual disability, language impairment, and self‐harm, although the rate of EEG abnormalities associated with ASD varies from many reports. However, recent brain function and genetic studies have suggested that ASD is associated with schizophrenia,^21^ and ADHD with and without ASD should be considered separately.

Several studies have been conducted on epileptic discharge in ADHD. According to one study, 5%‐60% of patients with ADHD demonstrate epileptic discharges, of whom 14% show transition to epilepsy.^22^ A study by Holtmann et al,^23^ which enrolled 48 children, including 16 children with ADHD and Rolandic spikes (RS), 16 children with ADHD without RS, and 16 control children, showed that RS were detected more frequently in children with ADHD who demonstrated higher impulsivity. In addition, in children with ADHD and RS, RS were detected more frequently in children with ADHD and higher impulsivity. Similarly, children with ADHD and RS showed decreased focus and interference control compared with the control group. Furthermore, sleep polysomnography conducted in 42 children with ADHD showed that 53.1% of children developed epileptic discharges.^24^ Hence, it is necessary to consider the possibility of exacerbation of epileptic seizures and EEG abnormalities caused due to central nervous system (CNS) drugs in ADHD treatment. Therefore, we thought that we should consider whether epileptic discharges, not basic wave abnormalities, are affected by ADHD treatments. There is a previous study^25^ that elucidates EEG abnormalities in ADHD by analyzing the appearance time of epileptic discharges. We referred their study method, called PI index, in this study.

Methylphenidate (MPH), a commercially available CNS stimulant used to treat ADHD in Japan, has been reported to lower seizure threshold. However, there have been no comparative studies on EEG changes before and after the administration of ADHD treatment drugs, including atomoxetine (ATX), a non‐CNS stimulant. Therefore, we examined the changes in sleep EEGs before and after the administration of anti‐ADHD medication.

## 2 METHODS

The medical records of patients who gave consent were retrospectively examined with the approval of the Ethics Committee of NCNP (approval number A2014‐114). Paroxysmal abnormalities were obtained from sleep EEGs, assuming that the record of sleep stages 1‐2 occurred for at least 10 minutes. EEG recordings were performed in all patients under sedation with oral medication, pentobarbital, or triclofos sodium. No complications were observed by sedation in all patients.

**FIGURE 1:**
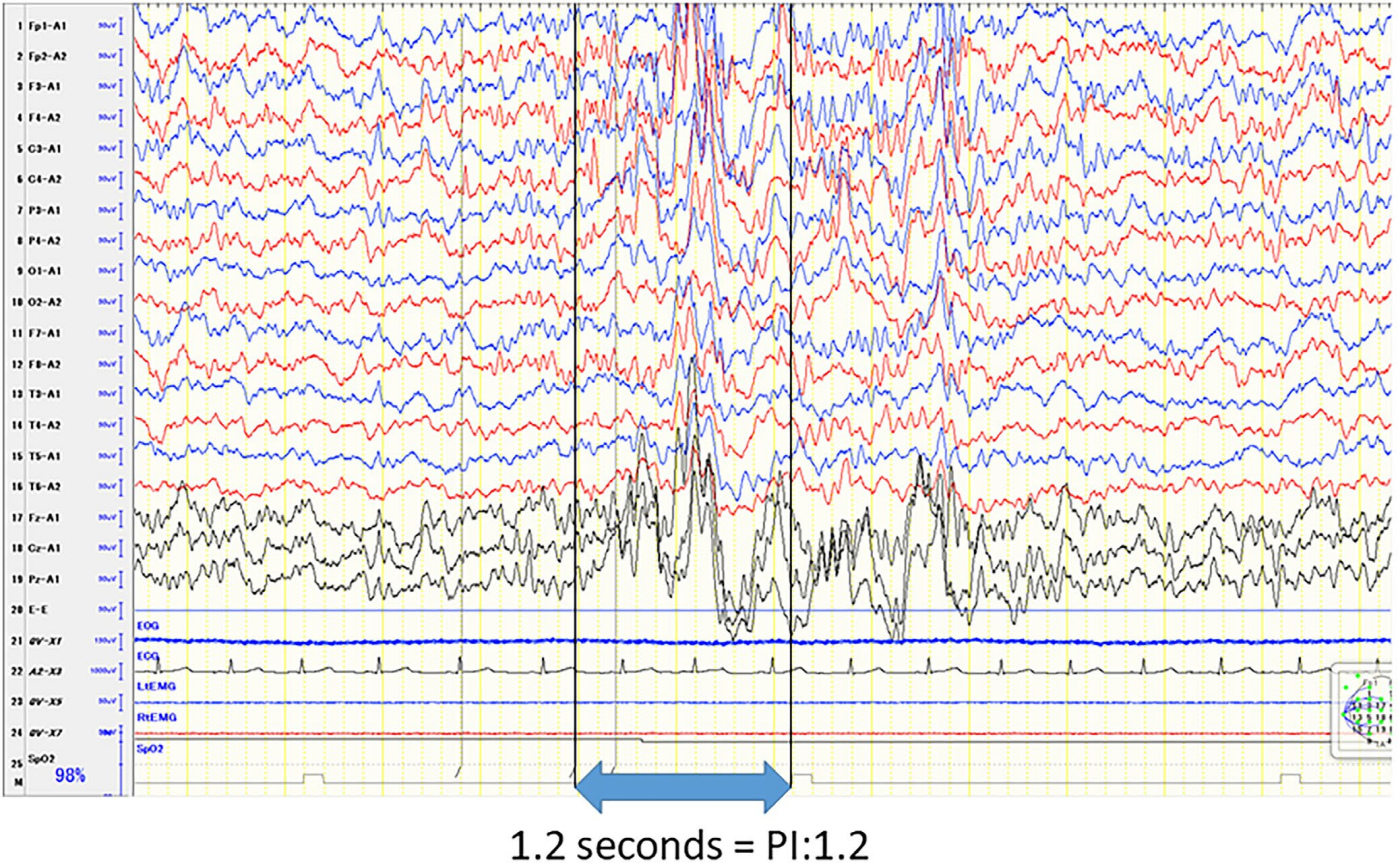
Paroxysmal index: PI (referential derivation). Above is shown for example of PI. PI was calculated epileptic discharge occurrence duration

Electroencephalography assessment was performed by measuring spikes, slow waves, and spike‐and‐wave complexes detected in a 10‐minute EEG recording during light sleep in 0.1‐second units, as previously described by Altunel et al^25^ The wave durations were calculated as the paroxysmal index (PI; Figure 1). PI was calculated visually by a pediatric neurologist. The PIs were then compared before and after the oral administration of ADHD drugs. In addition, the ADHD Rating Scale (ADHD‐RS) and Clinical Global Impression‐Improvement (CGI‐I) scale were distributed to parents to evaluate the clinical symptoms of ADHD; similarly, the changes before and after the oral administration of ADHD drugs based on these scales were compared with the PIs in patients who responded to the questionnaires.

Statistical analysis was performed using the Wilcoxon signed‐rank test to detect PI changes before and after treatment. PI changes before and after treatment were evaluated using the *t* test in each of the MPH and ATX groups. The *U* test was used with or without epilepsy and the change in PI before and after treatment.

Among those who visited our epilepsy/developmental disorder outpatient clinic in the National Center of Neurology and Psychiatry (NCNP) hospital between March 2011 and April 2016, the following patients were included: (a) those diagnosed with ADHD by certified pediatric neurologists using the Diagnostic and Statistical Manual of Mental Disorders, 4th Edition, Text Revision (DSM‐4‐TR) or DSM‐5; (b) those who underwent sleep EEG before and after oral MPH or ATX administration; (c) those in whom there had been no changes in antiepileptic drugs (AEDs) due to seizures; and (d) those in whom there had been no changes in MPH and ATX dose. Other clinical characteristics data were collected from medical records: epilepsy, ASD complications, and drug history. Patients with incomplete EEG records what was <10 minutes or without sleep state were excluded.

## 3 RESULTS

### 3.1 Clinical findings

We included 26 patients (21 men and 5 women) but finally performed the analysis in 28 patients (23 men and 5 women) because two patients were taking MPH and ATX at different times, they were included in this study (Table 1). A total of 18 patients (16 men and 2 women) received oral MPH, and 10 (7 men and 3 women) received oral ATX. The mean age of the patients at the start of MPH or ATX was 10.2 ± 2.0 (mean ± standard deviation) years; the mean age of epilepsy patients only was 10.5 ± 2.0 (mean ± standard deviation) years. The mean age at the time EEG evaluation of the patients before the start of oral administration of ADHD treatment drugs was 10 8 ± 2.7 (mean ± standard deviation) years, and the mean age at the time EEG evaluation of after the start of that was 11.2 ± 2.8 years. EEG evaluation was performed at intervals of a minimum of 3 months to a maximum of 4 years (1.2 years on average) before and after oral administration. The duration of therapy ranged from 3 to 57 months, with a mean interval of 14.3 ± 10.9 months. None of the patients received both drugs simultaneously. Comorbid epilepsy was present in 10 patients (7 in the MPH group and 3 in the ATX group), and 8 patients (5 in the MPH group and 3 in the ATX group) had comorbid ASD. Epilepsy and ASD were diagnosed prior to the diagnosis of ADHD in all cases. ADHD‐RS and CGI‐I were completed in 24 patients. Moreover, three patients did not receive AEDs throughout the study. The oral medications administered before the start of the evaluation were valproate (VPA), carbamazepine (CBZ), clobazam (CLB), lamotrigine, clonazepam, zonisamide, Chinese herbal medicine (Yokukansan), and levetiracetam (Table 2). In addition, nine patients were taking AEDs to improve the emotional aspects of their ADHD (VPA in six, CBZ in two, and CLB in one patient). In patients with epilepsy who were receiving AEDs, they were already being administered prior to ADHD treatment. In patients with comorbid epilepsy, no exacerbation of epileptic seizures was observed. These nine patients were started on AEDs after the initial EEG recording. On the other hand, none of the patients with epilepsy had added or changed the amount of AEDs before and after the administration of anti‐ADHD drugs.

**TABLE 1:** Characterization of cases

	MPH	ATX	Total
Number	18	10	28
Male:Female	16:2	7:3	23:5
Epilepsy	7	3	10
Autism spectrum syndrome	5	3	8
Interval of EEG analysis			
<6 mo	0	3	3
6 mo ≤ <12 mo	2	6	8
12 mo ≤ <24 mo	6	8	14
24 mo	2	1	3

Abbreviations: ATX, Atomoxetine; EEG, electroencephalography; MPH, methylphenidate.

**TABLE 2:** Medication of except for ADHD treatment

Medication	Continuance before pre‐treatment (n)	Add on post‐treatment (n)	Add on simultaneously with MPH (n)
VPA	6	4	1
CBZ	9		1
CLB	6		
Yokukansan	3	1	
LTG	3		
CZP	2		
ZNS	2		
LEV	1		

Note

No medication except MPH or ATX in whole period was three cases. Five cases were added medication post‐treatment.

Abbreviations: CBZ, Carbamazepine; CLB, clobazam; CZP, clonazepam; LEV, levetiracetam; LTG, lamotrigine; VPA, valproic acid; ZNS, zonisamide.

### 3.2 PI values

#### 3.2.1 Changes before and after drug administration

**FIGURE 2:**
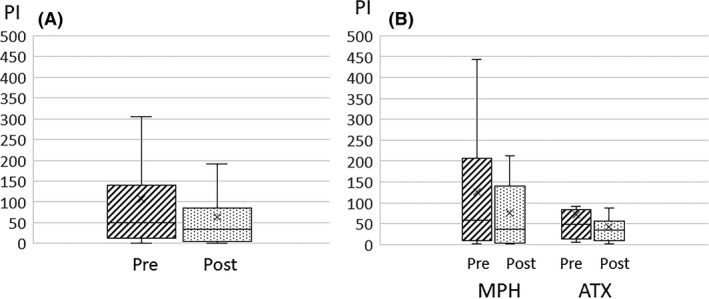
PI changes pre‐ and post‐ADHD treatment. Compared PI change with pre‐ and post‐ADHD treatment in all cases (A), in divided group MPH and ATX (B). No significant differences were seen. The cross marker showed average value. ADHD, attention‐deficit hyperactivity disorder; ATX, atomoxetine; MPH, methylphenidate; PI, paroxysmal index

The duration of abnormal waves tended to decrease after MPH and ATX administration (*P <* 0.06). A comparison of changes in the PI values in the MPH and ATX groups showed no significant differences (*t*(26) = 0.7, *P* = 0.49) (Figure 2).

**FIGURE 3:**
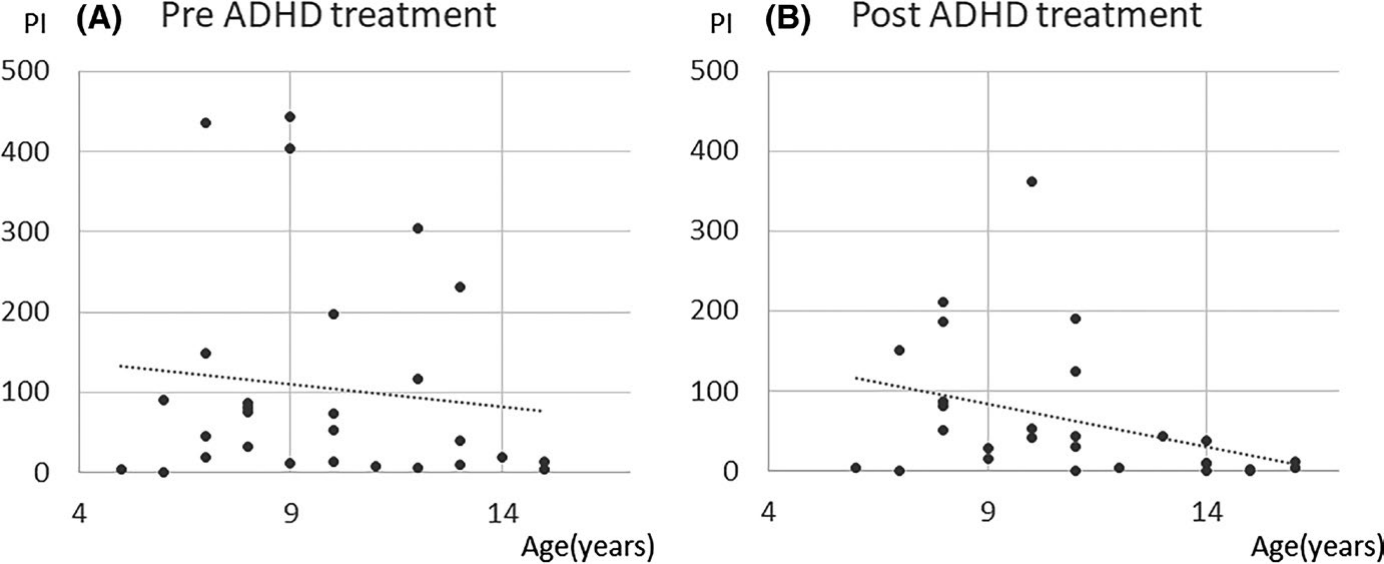
Correlation between PI change and age. Negative correlation with PI change and age was seen. The duration of epileptic discharges at post‐ADHD treatment (B) was less than pre‐ADHD treatment (A) in elder children. ADHD, attention‐deficit hyperactivity disorder; PI, paroxysmal index

**FIGURE 4:**
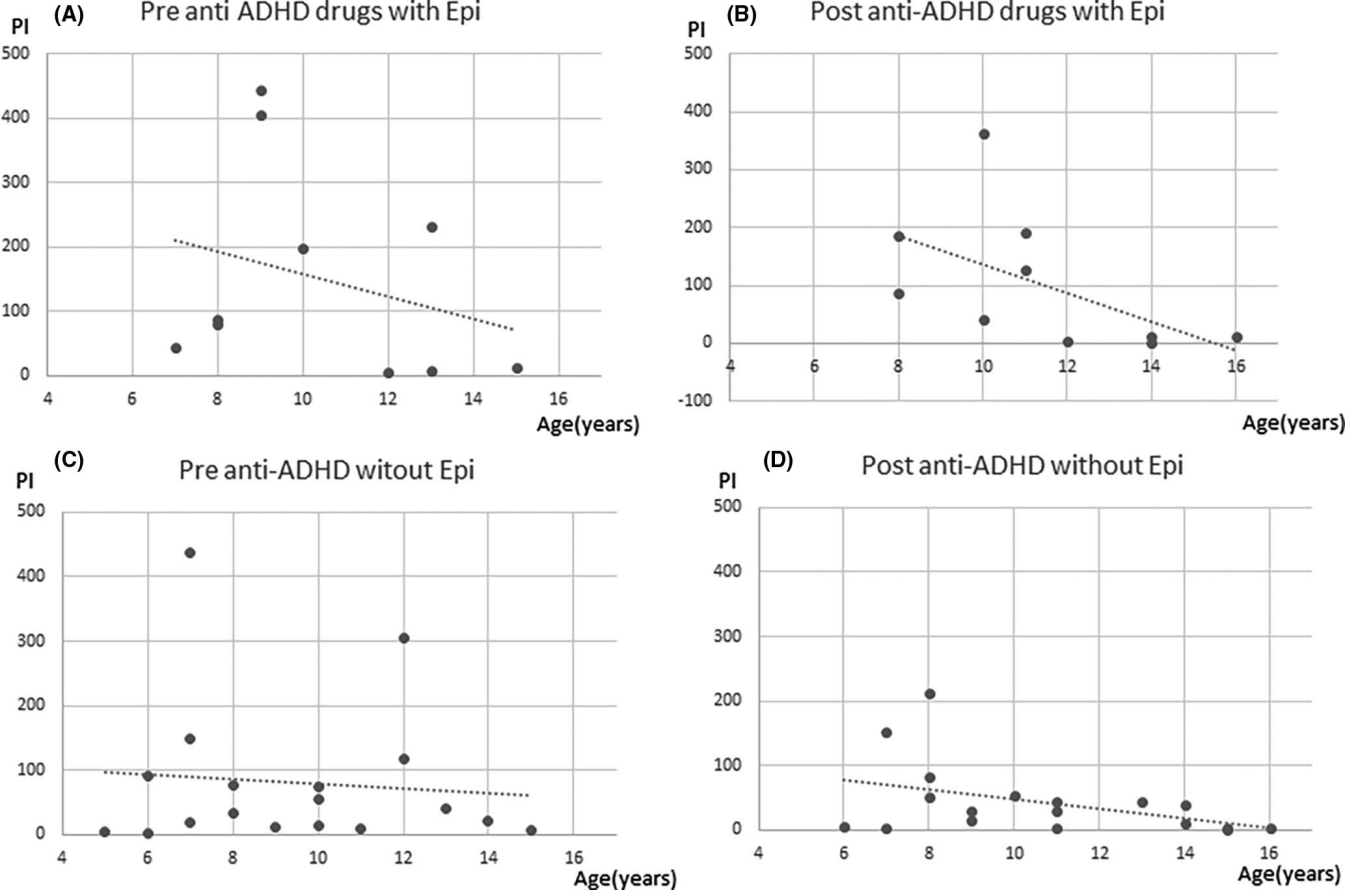
Correlation between PI change and age with and without epilepsy. The duration of epileptic discharges at post‐ADHD treatment (B) was less than pre‐ADHD treatment (A) in elder children with epilepsy. No significant correlation changes in PI values with patient age at the first and second in patients without epilepsy (C, D). ADHD, attention‐deficit hyperactivity disorder; PI, paroxysmal index

**FIGURE 5:**
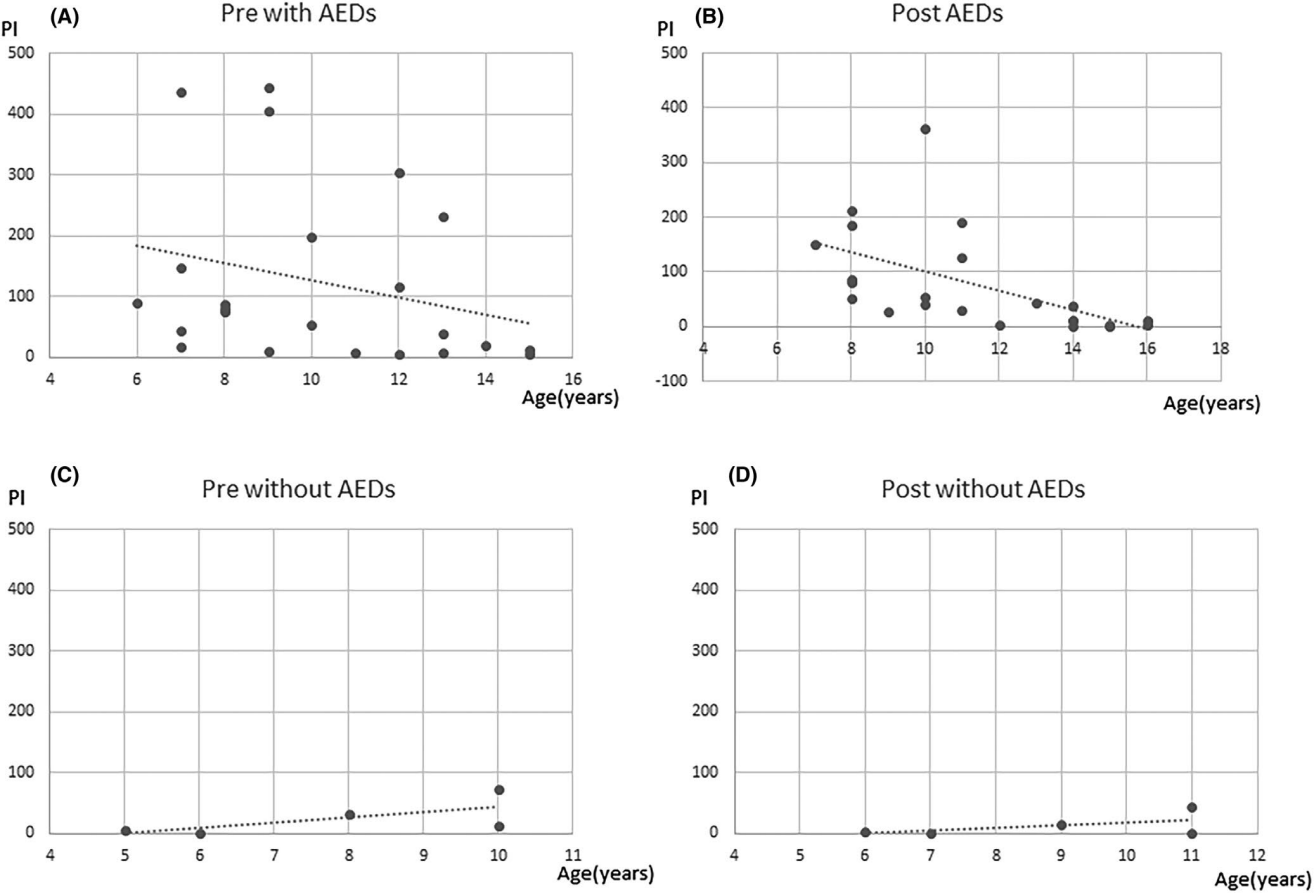
Correlation between PI change and age with and without AEDs. The duration of epileptic discharges at post‐ADHD treatment (B) was less than pre‐ADHD treatment (A) in elder children with AEDs. No significant correlation changes in PI values with patient age at the first and second in patients without AEDs (C, D). ADHD, attention‐deficit hyperactivity disorder; AED, antiepileptic drugs; PI, paroxysmal index

**FIGURE 6:**
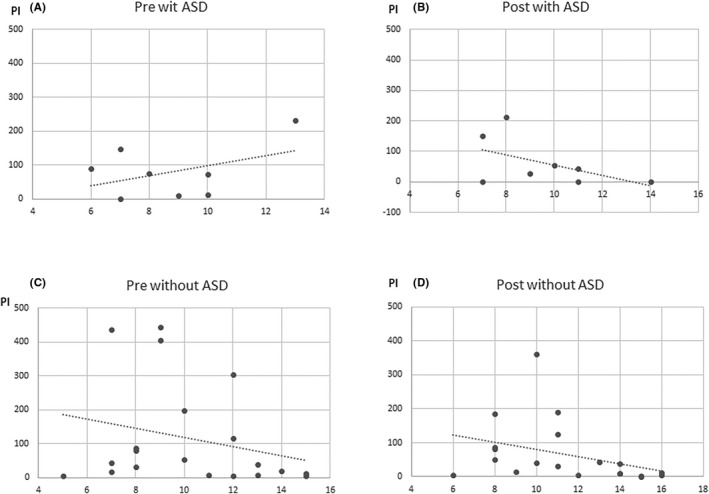
Correlation between PI change and age with and without ASD. The duration of epileptic discharges at post‐ADHD treatment (D) was less than pre‐ADHD treatment (C) in elder children without ASD. No significant correlation changes in PI values with patient age at the first and second in patients with AED (A, B). ADHD, attention‐deficit hyperactivity disorder; AED, antiepileptic drugs; ASD, autism spectrum disorders; PI, paroxysmal index

Changes in PI values showed a significant negative correlation with patient age (age at the first measurement: Spearman’s *ρ* = −0.44, *P =* 0.018; age at the second measurement: Spearman’s *ρ* = −0.52, *P* = 0.003), indicating older patients had longer durations of abnormal wave decreases after administration (Figures 3, 4, 5, 6).

Similarly, changes in PI values with epilepsy showed a significant negative correlation with patient age (age at the first measurement: Spearman’s *ρ* = −0.26, *P =* 0.462; age at the second measurement: Spearman’s *ρ* = −0.65, *P* = 0.041). There were no significant correlation changes in PI values with patient age at the first and second in patients without epilepsy.

Changes in PI values administrated AEDs showed a significant negative correlation with patient age (age at the first measurement: Spearman’s *ρ* = −0.39, *P =* 0.065; age at the second measurement: Spearman’s *ρ* = −0.75, *P* < 0.0001). Patients not administrated AEDs were too small number, so that there was no indication to do statics.

Changes in PI values with ASD showed no significant negative correlation with patient age (age at the first measurement: Spearman’s *ρ* = 0.08, *P* = 0.842; age at the second measurement: Spearman’s *ρ* = −0.56, *P* = 0.143). In patients without ASD, changes in PI values showed a significant negative correlation with patient age (age at the first measurement: Spearman’s *ρ* = 0.26, *P =* 0.260; age at the second measurement: Spearman’s *ρ* = −0.56, *P* = 0.009).

#### 3.2.2 Comorbid epilepsy and PI values

**FIGURE 7:**
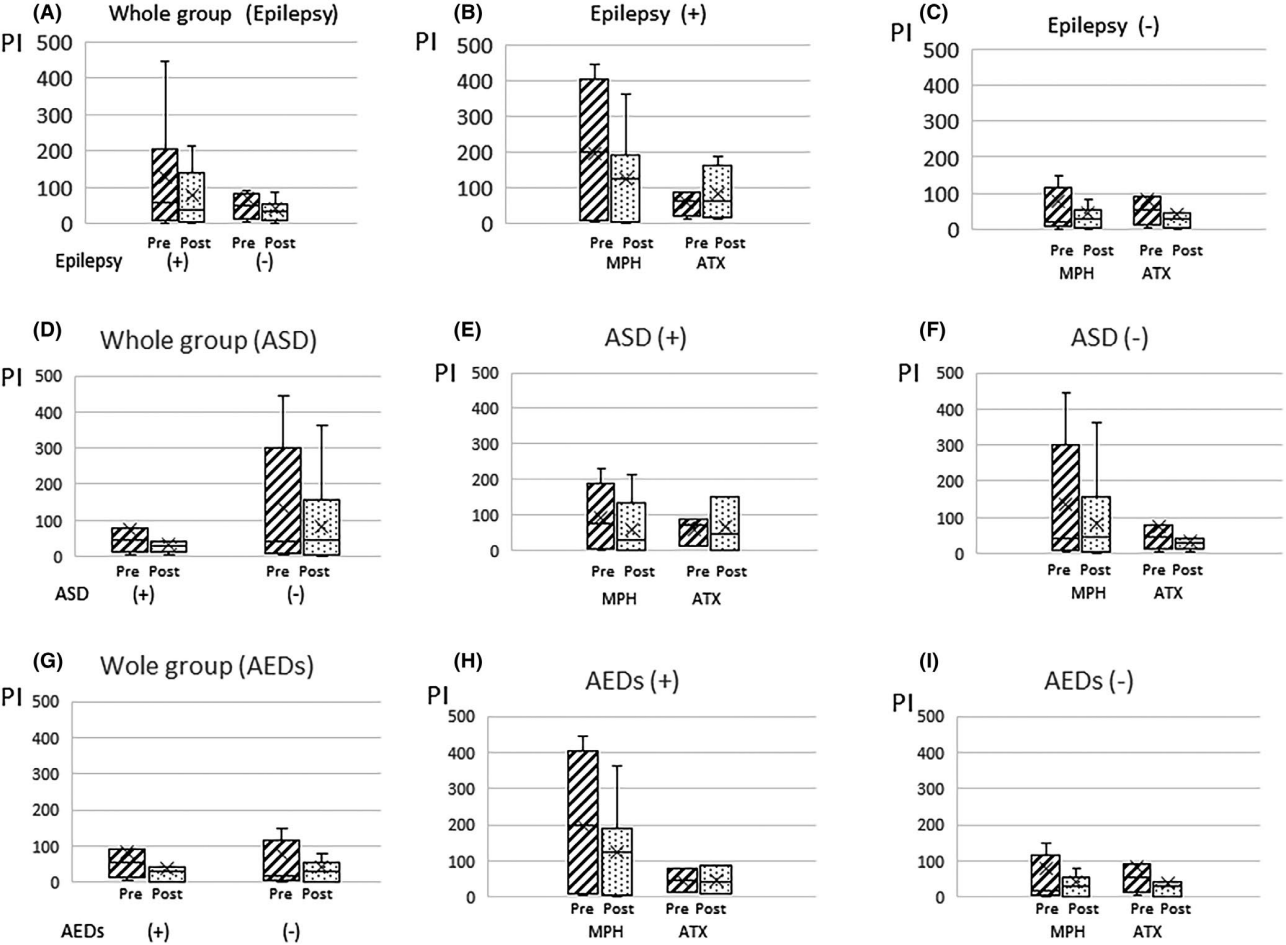
PI change of pre‐ and post‐ADHD treatment with or without epilepsy. Compared with PI change of pre‐ and post‐ADHD treatment with or without epilepsy (A), MPH and ATX group with epilepsy (B), MPH and ATX group without epilepsy (C), with or without ASD (D), MPH and ATX group with ASD (E), MPH and ATX group without ASD (F), with or without AEDs (G), MPH and ATX group with AEDs (H), MPH and ATX group without AEDs (I). PI change was no significant difference whether with or without epilepsy, ASD, and AEDs. The cross marker showed average value. ADHD, attention‐deficit hyperactivity disorder; AED, antiepileptic drugs; ASD, autism spectrum disorders; ATX, atomoxetine; MPH, methylphenidate; PI, paroxysmal index

No difference was observed in the changes in PI values between patients with comorbid epilepsy and those without epilepsy (*P* = 0.40) (Figure 7). In addition, there was no significant difference in the changes in PI values between the MPH and ATX groups in patients with comorbid epilepsy and those without epilepsy.

There was no difference in the changes in PI values between patients with comorbid ASD and those without ASD (*P* = 0.56), and whom of AEDs administration and without AEDs (*P* = 0.86). Between the MPH and ATX groups in patients with or without comorbid ASD and those administration or no administration AEDs had also no significant difference in the changes in PI values between the MPH and ATX groups.

#### 3.2.3 ADHD‐RS, CGI, and PI values are shown in Figures 8 and 9

**FIGURE 8:**
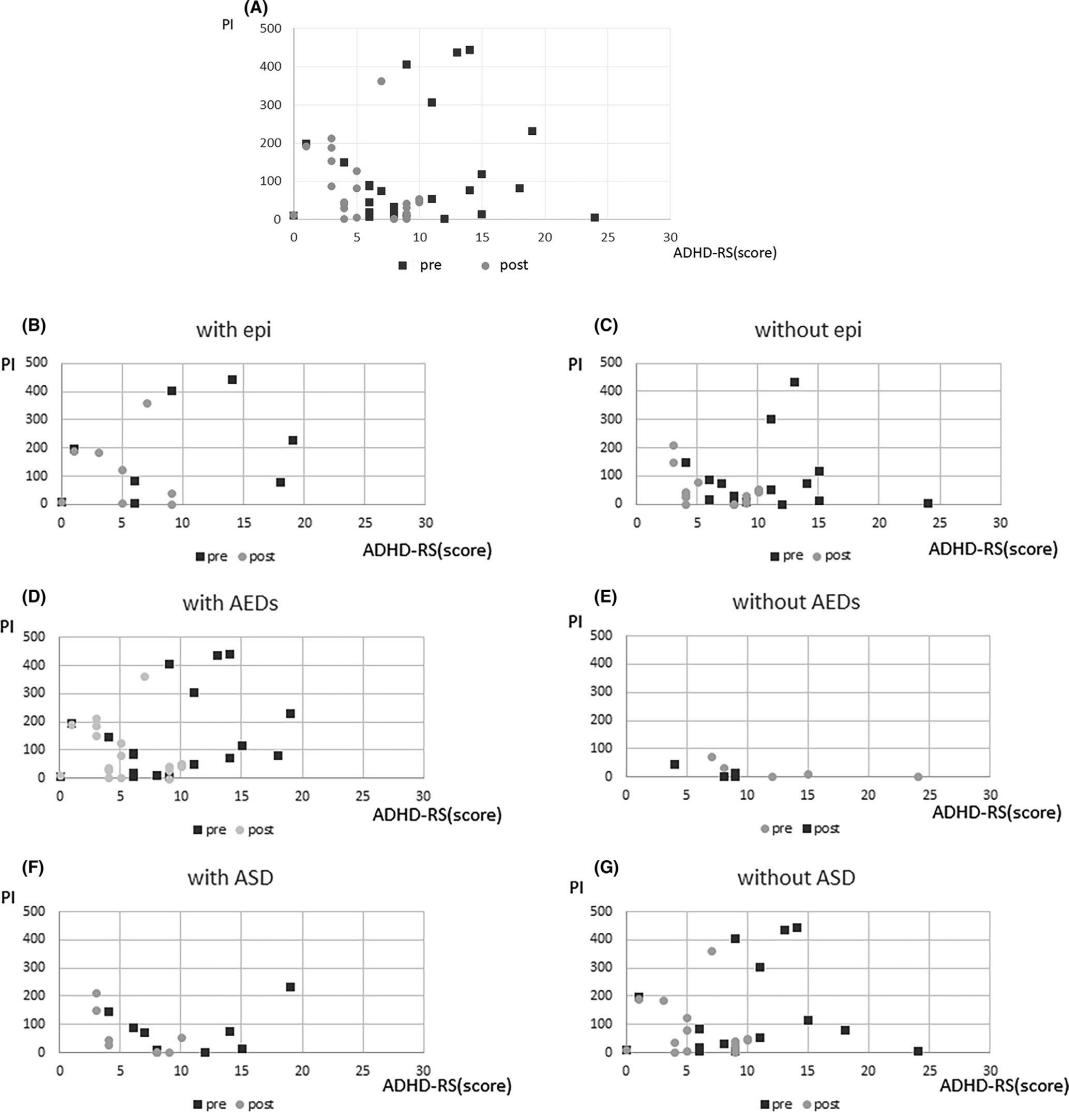
PI change and ADHD‐RS. PI decrease and reduction of hyperactivity were negative significant correlation (A) (*ρ* = 0.49, *P =*0.032). No significant correlation PI change and hyperactivity of ADHD‐RS whether epilepsy, AEDs, ASD or not (B‐G). ADHD, attention‐deficit hyperactivity disorder; ADHD‐RS, ADHD Rating Scale; AED, antiepileptic drugs; ASD, autism spectrum disorders; PI, paroxysmal index

**FIGURE 9:**
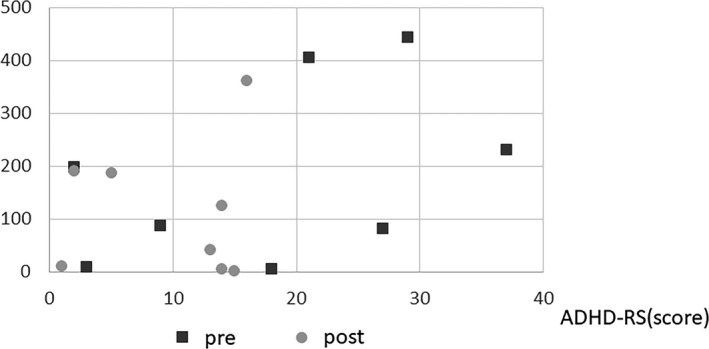
PI change and score of ADHD‐RS (with epilepsy group only). PI decrease and ADHD symptoms were negative correlation in epilepsy. (Spearman’s *ρ* = 0.74, *P* = 0.035). ADHD, attention‐deficit hyperactivity disorder; ADHD‐RS, ADHD Rating Scale

For the ADHD‐RS, the difference in values before and after oral administration was used to signify the change in symptoms (Figures 8 and 9). Overall, the decrease in PI values was significantly correlated with hyperactivity (*ρ* = 0.49; *P =* 0.032), but there was no correlation with inattention. In particular, the MPH group showed a significant correlation between the decrease in PI values and hyperactivity (*ρ* = 0.50; *P* = 0.048), whereas the ATX group did not show any significant correlation. There was no significant correlation between the PI values and each item of the ADHD‐RS in both patients with and without comorbid ASD. Similarly, there was no significant correlation between patients administrated AEDs and those not administrated AEDs.

Patients with comorbid epilepsy showed a significant correlation between the PI values and each item of the ADHD‐RS (inattention: *ρ* = 0.74; *P* = 0.035; hyperactivity: *ρ* = 0.71; *P* = 0.050), and a significant correlation was also observed with the total score (*ρ* = 0.74; *P* = 0.035). However, patients without epilepsy showed no significant correlation between PI values and any item of the ADHD‐RS.

The PI values and CGI‐I scores showed no correlation in any analysis.

## 4 DISCUSSION

The EEGs of patients with ADHD showed basic wave and sporadic abnormalities that were investigated in this study. Another study showed that MPH administration normalized the EEG in 56.9% of patients, with no change in 33.8%, and worsened the EEG in 9.3% of patients, indicating an inconsistency in EEG changes due to ADHD treatment drug administration.^26^ However, another study indicated that in cases where the EEG was normalized, there was a decrease in θ waves and an increase in β waves.^27^ In addition, there are few reports on the changes in basic waves due to ATX administration, with one report demonstrating an increase in β waves.^28^ In the present study, the older the patient, the more significant the decrease in the duration of abnormal waves. No reports have yet investigated the effects of ADHD treatment drugs on sleep EEGs; however, the changes could be due to cerebral maturation, as is the case with basic waves. Because no significant differences were observed in the PI changes in either the MPH or ATX group, it was considered that there were no adverse effects, at least on the abnormal sleep EEG.

Methylphenidate has been used to lower the seizure threshold. However, some recent studies have revealed that MPH administration does not exacerbate epilepsy in patients with epilepsy; that is, MPH administration does not increase the frequency of seizures in patients with epilepsy and ADHD.^29^, ^30^ Furthermore, there have been relatively few reports on ATX administration in patients with epilepsy. Torrs et al^31^ reported the tolerability of ATX in patients with epilepsy; there were no discontinuations of ATX due to epileptic seizure exacerbation. The results of the present study showed that there were no effects on the PI changes in the ATX group, and comorbid epilepsy had no impact on PI changes. This result is consistent with that of a previous report.^31^ Because no exacerbation of epileptic seizure was observed in any of the cases of comorbid epilepsy, it was considered that the addition of ADHD medication did not exacerbate EEG abnormalities or seizures due to oral AEDs in patients with comorbid epilepsy.

This research studied paroxysmal abnormalities on EEG before and after ADHD treatment drugs. Total 19 patients had been administrated AEDs; thus, it was possible EEG abnormalities were decreased by the administration of AEDs. There are several previous studies on the effect of AEDs on EEG findings. Libenson et al^32^ reported the rate of clearance of interictal epileptiform activity before and after AEDs treatment. They showed VPA superior to Phenobarbital in suppressing interictal epileptiform activity, including both focal and generalized epileptiform activity. Other report described AEDs improve sleep architecture, decrease sleep latency and nocturnal awakening which indirectly may modify interictal EEG abnormalities.^33^ Spencer et al^34^ confirmed a small but significant decrease in interictal discharges by AED withdrawal. Another study reported some AEDs could abolish ictal epileptiform discharge in vitro, but that was unable to block interictal activity on EEG.^35^ These authors concluded interictal activity is unaffected by AED levels that are effective to stop seizures, that drug levels that influence seizure occurrence do not influence interictal discharge rate. For these reasons, the effect of AEDs on EEG findings cannot be denied, but we thought that AEDs did not completely improve EEG abnormalities between seizures.

In the present study, we investigated the correlation between ADHD symptoms and PI using a questionnaire administered to patients. The results revealed that there was a correlation between decreased PI values and improved hyperactivity in the MPH group, although there was no correlation between decreased PI and hyperactivity in the ATX group. While there was a difference between the two drugs, (MPH is a CNS agent, while ATX is a non‐CNS agent), both MPH and ATX improve inattention and hyperactivity/impulsivity. One study showed that MPH improved hyperactivity, learning disability symptoms, and heat‐induced seizures in epileptic rats,^36^ and another study showed that MPH improved ADHD symptoms in 75% of patients with epilepsy.^37^ Thus, the results of the present study are consistent with those of previous studies on MPH.

Regarding ATX and changes in ADHD symptoms and EEG basic waves, Chiarenza et al^38^ reported the results of their investigation in which subjects were divided into ATX responders and non‐responders using the Swanson, Nolan, and Pelham (SNAP‐IV) rating scale. They reported that α waves increased in the frontal and temporal regions in the ATX responder group, and all frequency bands increased in the non‐responder group. Thus, we concluded that the difference in EEG changes between MPH and ATX may be due to the possibility that ATX affects basic and sporadic wave abnormalities.

Eight patients with ASD were included in present study. There were no significant PI changes between with and without ASD before and after ADHD treatment. There were a significant correlation changes in PI values and patient age with ASD; however, there was no significant correlation that without ASD in our study. This suggests that ASD is more likely to be complicated by EEG abnormalities, but that there is little improvement with age. Nicotera et al^20^ suggested EEG abnormalities and behavioral abnormalities were correlated in ASD patients. On the other hand, Luz‐Escamilla et al^32^ described in the absence of reports that prove an association between IED and ASD, patients should not be subjected to expensive treatments, such as the administration of anticonvulsant therapies. We only investigated the presence of autism complications, had no research on the relationship between symptoms and EEG abnormalities in this study. Hyperactivity may also be observed in ASD and should be additionally investigated for questionnaires in future.

The first limitation of this study was its single institution design and subsequent small sample size, which may have introduced inherent bias. In Japan, MPH, which can be prescribed only by certified doctors under the strict control of the Ministry of Health, Labor, and Welfare, has been carefully administered to patients with ADHD and epilepsy, meaning that relatively few patients could be examined at our institution. In addition, EEG to measure sleep state is not essential when starting ADHD treatment, and it was difficult to collect many cases because some children underwent only awake EEG. Second, there was no consideration of the developmental effects on EEG changes. Previous studies have reported that changes in the basal wave distribution occur during development from children to adults.^11^ Third, we did not investigate the other two ADHD treatments in the Japanese market (Guanfacine and Lisdexamfetamine mesylate), as they had not been released at the time of this study. Fourth, patients who visited our clinic after the diagnosis of epilepsy were included. Because of this, we were not able to compare EEG abnormalities before and after AEDs medication. Fifth, we were not able to investigate the relationship between the duration of MPH or ATX administration and EEG findings. In addition, although there was no change in the dosage of AEDs, the effect of the duration of administration on EEG findings was not fully investigated, and the timing of EEG measurement for ADHD drugs varied from case to case, which may have affected the results.

In this study, we considered that there were no adverse effects on the abnormal sleep EEG in ADHD patients following administration of ADHD treatment, especially MPH. Although there was a concern that MPH administration in patients with ADHD with a history of epilepsy would exacerbate seizures, the results of this study suggest that it can be safely administered to patients with ADHD symptoms. For patients with ADHD, alleviating ADHD symptoms is also involved in quality of life^39^; therefore, appropriate treatment is desired even for patients with ADHD with epilepsy.

## CONFLICT OF INTEREST

Author EN has received speaker honoraria from Eisai Co., Ltd. The other authors declare no competing interests.

## AUTHOR CONTRIBUTIONS

HY, EN, YKita, and MI were involved in study design and data interpretation. HY, Kita, and MI were involved in the data analysis. All authors critically revised the report, commented on drafts of the manuscript, and approved the final report.

## APPROVAL OF THE RESEARCH PROTOCOL BY AN INSTITUTIONAL REVIEWER BOARD

Reviewer Board: approval of the Ethics Committee of NCNP (approval number A2014‐114).

## Data Availability

The data that support the findings of this study are available from the corresponding author upon reasonable request.
